# Ambient air pollution and the sex ratio at birth: a systematic review and narrative synthesis

**DOI:** 10.1101/2025.10.21.25338487

**Published:** 2025-10-23

**Authors:** Ursula Gazeley, Hallie Eilerts-Spinelli, Jasmin Abdel Ghany, Ana Bonell, Joshua Wilde

**Affiliations:** 1 Leverhulme Centre for Demographic Science, Nuffield Department of Population Health, University of Oxford, Oxford, United Kingdom; 2 Nuffield College, University of Oxford, Oxford, United Kingdom; 3 International Health, Johns Hopkins Bloomberg School of Public Health, Baltimore, Maryland, United States; 4 Medical Research Council Unit The Gambia at London School of Hygiene and Tropical Medicine, Fajara, The Gambia; 5 Portland State University, Portland, Oregon, United States

**Keywords:** Air pollution, sex ratio at birth, in utero exposure, conception, stillbirth, miscarriage, pregnancy loss

## Abstract

Ambient air pollution is associated with adverse pregnancy outcomes such as stillbirth, miscarriage, and preterm birth. However, its effects on the viability, implantation, and survival of conceptuses—largely unobserved before the clinical detection of pregnancy—remain poorly understood. Shifts in the sex ratio at birth (SRB) provide a sensitive, population-level indicator of sex-biased conception and pregnancy loss. This systematic review examined whether maternal exposure to ambient air pollution before conception and during pregnancy is associated with deviations in the SRB. We searched MEDLINE, Embase, and Global Health from inception to 12 February 2025 for observational studies assessing both ambient air pollution exposure and SRB. Eleven studies met the inclusion criteria, representing data from eight countries spanning 1970–2023. PM_10_ was the most frequently investigated pollutant, followed by PM_2.5_. Six studies reported no significant association, while four observed increased feminisation with higher exposure levels. Air pollution may plausibly influence SRB through effects on gamete quality, conceptus viability, and placental development. The SRB therefore provides a valuable proxy for detecting early biological vulnerability, revealing effects of air pollution that are otherwise hidden due to the selective observation of clinically recognised pregnancies. However, current evidence remains limited, heterogeneous, and inconclusive. As ambient air quality continues to deteriorate, more standardised research is needed to improve comparability and quantify the impact of air pollution on reproductive outcomes.

## 1. Introduction

Ambient air pollution has been consistently associated with adverse pregnancy outcomes, including miscarriage and stillbirth, and adverse perinatal outcomes, such as preterm birth, small for gestational age (SGA), and low birth weight (LBW) (Nyadanu et al., 2022; Song et al., 2023). These effects are well established in both high-income and low- and middle-income countries (LMICs). However, these observed outcomes represent only a selective subset of pregnancies that survive beyond clinical recognition to later gestational ages. (Wilcox et al., 1988; Ammon Avalos, Galindo and Li, 2012). As a result, much of the impact of air pollution exposure on conception, implantation, and early embryonic development remains unmeasured.

Many, if not most, human conceptions do not end in a live birth (Wilcox et al., 1988; Ammon Avalos, Galindo and Li, 2012). Early pregnancy losses occurring before clinical recognition are typically unobserved or unrecorded outside of assisted reproduction. These losses can be inferred at the population-level using the sex ratio at birth (SRB) – defined as the ratio of male to female live births (Bruckner and Catalano, 2018; Catalano, Casey and Bruckner, 2020). In the absence of sex-selective induced abortion, the natural human SRB is typically considered to fluctuate around 103-105 males per 100 live born females (Ein-Mor et al., 2010; Chao et al., 2019). However, evidence suggests that acute or chronic maternal stressors can reduce this ratio, leading to more female live births than expected (Bruckner and Catalano, 2018; Catalano, Casey and Bruckner, 2020). Deviations from the baseline SRB may arise through two, potentially co-occurring pathways: (1) sex-biased conception, which alters the likelihood of conceiving a male versus female zygote; and (2) sex-biased pregnancy loss, including failures at implantation and differential survival of male versus female conceptuses in utero, including miscarriage and stillbirth. Identifying changes in the SRB associated with maternal exposures can therefore serve as a useful proxy for otherwise unobserved changes in reproductive health.

A range of environmental exposures – including extreme heat stress (Abdel Ghany et al., 2024), cold stress (Catalano, Bruckner and Smith, 2008), water pollution (Long et al., 2021), exposure to toxins such as PCBs, dioxins, pesticides, and lead (Terrell, Hartnett and Marcus, 2011; Azizi et al., 2024) – have been associated with reductions in the SRB, resulting in a skew towards female live births. Biological mechanisms remain unclear, but endocrine disrupting toxins may affect the sex ratio at conception via the production, viability, or motility of Y-bearing sperm (James, 2001). After conception, male conceptuses may be more susceptible to environmental stressors due to faster growth rates, higher metabolic demands, and less resilient placental function, making them more vulnerable under adverse conditions (Clifton, 2010; Saoi et al., 2020). Consistent with the Trivers-Willard hypothesis (Trivers and Willard, 1973)— that female offspring have higher reproductive success than males when maternal condition is poor—male-biased vulnerability in utero offers a plausible evolutionary explanation for observed SRB shifts.

**Figure 1.**
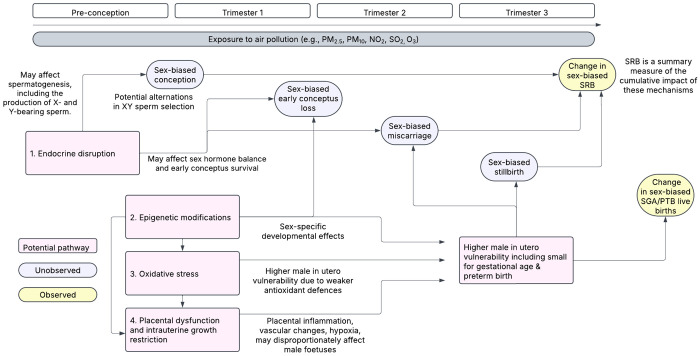
Conceptual framework of potential mechanisms of air pollution exposure on the sex ratio at birth

While the independent effects of air pollution on pregnancy outcomes, and of acute maternal stressors on the SRB, are relatively well documented, it remains unclear whether air pollution exposure contributes to shifts in the SRB at the population-level (Terrell, Hartnett and Marcus, 2011). Conceptually, several plausible pathways through which air pollution could affect the SRB are illustrated in Figure 1. To date, however, no systematic review has focused exclusively on ambient air pollution and the SRB. The only prior review considered environmental pollutants broadly, with air pollutants as a subset, and is now outdated (Azizi et al., 2024). A targeted, up-to-date synthesis is necessary to assess the specific contribution of air pollution to the SRB.

Change in the SRB could serve as a sentinel marker of reproductive health (Bruckner and Catalano, 2018; James and Grech, 2018), signalling an otherwise largely unobserved impact of exposure to ambient air pollution. Understanding these patterns is essential to assess the full reproductive and population-level impact of environmental exposures and to inform maternal health policy and air quality regulation. Moreover, changes in the SRB may also have long-term demographic and societal implications. Skewed SRBs can affect future partnering dynamics, family formation patterns, and population dynamics, which may only become apparent decades later (Guilmoto, 2009). Potential changes in the SRB resulting from ambient air pollution exposure may therefore extend beyond reproductive health to public policy more broadly.

Ambient air pollution is already an urgent public health issue, and its burden is expected to worsen. Nearly the entire global population already lives in areas that do not meet WHO air quality guidelines (World Health Organization, 2021). While mechanisms are complex, climate change may intensify the air pollution, increasing ground-level ozone formation and fuelling wildfires, which contribute to elevated particulate matter (World Health Organization, 2021). Co-exposure to heat and air pollution may also have synergistic effects on adverse pregnancy outcomes (Raghavan et al., 2025). Rapid urbanisation, particularly in LMICs, is exposing growing populations to worsening air quality. These worsening air quality conditions may amplify any existing effects on SRB, making it imperative to understand these relationships now. This systematic review aimed to assess the available evidence on the relationship between ambient air pollution and the SRB.

## 2. Methods

The review protocol is registered on PROSPERO (no. CRD420250651445).

### 2.1 Search strategy details

We conducted a systematic review in accordance with the PRISMA guidelines (appendix pp.2-3). We searched MEDLINE, Embase, and Global Health (Ovid) on 12 February 2025, without language or date restrictions, using a combination of controlled vocabulary (MeSH for MEDLINE, Emtree for Embase) with free text search (by title and abstract for Global Health; by title, abstract and keywords for MEDLINE and Embase (see appendix p.4 for the full search). Air pollution search terms included (1). Criteria air pollutants, e.g., particulate matter (PM_2·5_, PM_10_, ultrafine particles), nitrogen dioxide and oxides, sulphur dioxide, ozone, carbon monoxide; (2) Hazardous Air Pollutants (e.g., Volatile organic compounds, polycyclic aromatic hydrocarbons, heavy metals persistent organic pollutants, and airborne pesticides or endocrine disrupting chemicals; (3) Emerging air pollutants (e.g., black and brown carbon, and microplastics). Air pollution exposure terms were combined with sex ratio search terms (e.g., “sex ratio”, “male-to-female birth ratio”, “proportion of male births”, “probability of male birth”).

### 2.2 Inclusion and exclusion criteria

We included original observational and quasi-experimental studies, including cohort studies (both prospective and retrospective), case-control studies, and cross-sectional designs, that assessed exposure to ambient air pollution and the SRB in human populations. We excluded studies that were not original research studies, such as conference abstracts, letters, editorials or reviews. Systematic and scoping reviews were not included in the final analysis but were used to check for additional eligible studies through reference screening.

Eligible studies were those that examined ambient air pollution as the primary exposure, specifically measuring concentrations of air pollutants (e.g., PM_2·5_, PM_10_), regardless of whether these were of anthropogenic or biogenic origin. Studies that measured other pollution sources (e.g., indoor or occupational exposures) to adjust for confounding were eligible, but studies that focused exclusively on these non-ambient sources, without including estimates of ambient air quality, were excluded. Eligible studies measured maternal exposure during a defined pre-conception window, during pregnancy, or aggregated exposure and birth outcomes by calendar year. We excluded studies that did not measure ambient pollution concentrations but discussed air pollution as a potential explanation for changes in the SRB. We also excluded studies that assessed toxin exposure via biomarkers (e.g., blood serum, bone lead levels) that could not be temporally linked to pregnancy or a pre-conception window, but that rather reflect long-term cumulative exposure over the life course.

Studies that reported pregnancy outcomes (miscarriage, stillbirth) without assessing the sex ratio were excluded, as were those focusing solely on the primary (conception) sex ratio and paternal exposures (e.g., assisted reproduction studies examining spermatogenesis). Finally, we excluded in vitro fertilisation (IVF) studies as these do not reflect in utero exposure.

### 2.3 Screening and data extraction

Two reviewers independently screened results by title and abstract followed by full text review. Where conflicts arose, decisions were adjudicated by a third reviewer. Rayyan was used to screen all search records. For eligible studies, we extracted the following information from each study: year of publication, study location, study design, sample size (women or births), gestational exposure window, gestational measurement, residence criteria, AQ assessment, average exposure in sample, outcome measure, type of particulate matter, spatial resolution of air quality data, temporal frequency of air quality data, air quality modelling, and effect size. Data were double extracted by two reviewers, and any discrepancies were resolved by a third reviewer.

### 2.4 Risk of bias

Study quality was appraised using an adapted version of the NIH Quality Assessment Tool for Observational Cohort and Cross-Sectional Studies (NHLBI 2014), modified to accommodate ecological, quasi-experimental, and time-series designs. The adapted criteria evaluated study design, exposure and outcome assessment quality, control for confounding, statistical methods, and risk of bias (see appendix p.4). Two reviewers completed the quality appraisal, with discrepancies resolved by a third reviewer.

### 2.5 Data analysis

We used a narrative synthesis to summarise findings and an effect direction plot to show whether each study shows masculinisation, feminisation, or no change in the SRB with exposure to ambient pollution. This enabled a consistent presentation of effect direction across studies employing different outcome and effect measures. Meta-analysis was infeasible due to the limited number of eligible studies, combined with considerable heterogeneity in study design, pollutant types, exposure categorisations, pre-conception and pregnancy exposure windows, and spatial attribution (Valentine, Pigott and Rothstein, 2010).

## 3. Results

### 3.1 Study selection

**Figure 2.**
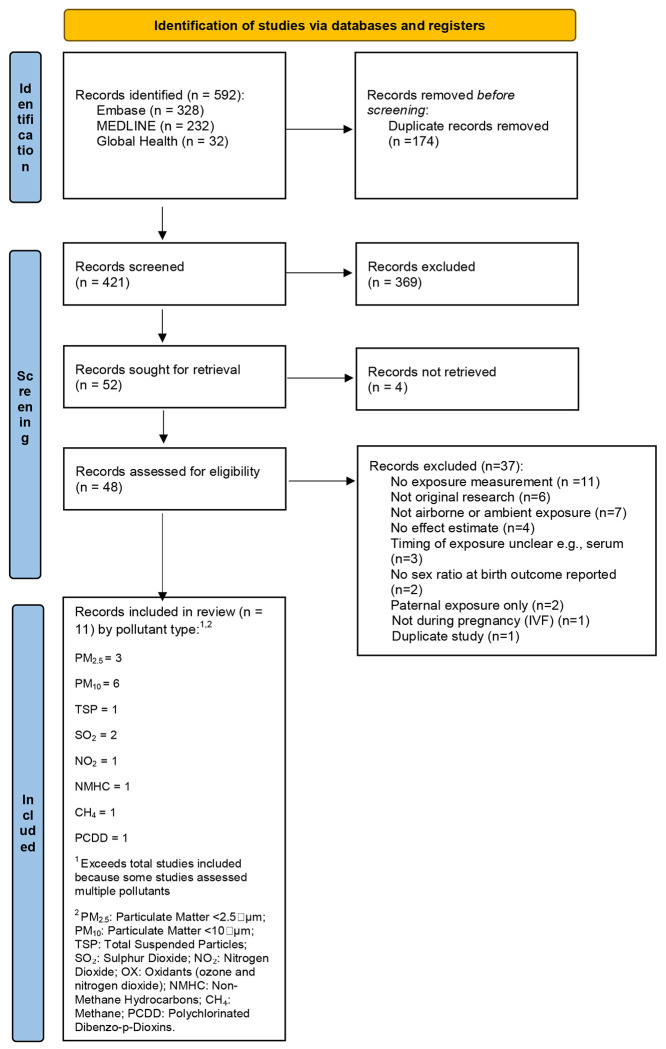
PRISMA flow diagram

The study selection process is illustrated in Figure 2 (PRISMA diagram). A total of 592 records were identified through database searches (Embase: 328; MEDLINE: 232; Global Health: 32). After removing duplicates, 421 unique records remained. Title and abstract screening reduced this to 52 studies selected for full-text review, of which 11 met the inclusion criteria.

### 3.2 Study characteristics

Table 1 presents the characteristics of the included studies. Of the 11 included studies, the years of observation spanned from 1970 to 2023, although most studies were published within the last decade. Sample sizes varied substantially, ranging from small cohorts to national-level datasets exceeding one million births (e.g., Great Britain (Ghosh et al., 2019, p. 20), USA (Sanders and Stoecker, 2015)). Several studies examined air quality for a population defined by their proximity to specific sources of ambient pollution, such as municipal waste incinerators (Lin, Li and Mao, 2006; Candela et al., 2013; Santoro et al., 2016; Ghosh et al., 2019). The evidence base was geographically narrow, with studies conducted in only eight countries, all of which were either high-income or upper-middle-income economies. Brazil and China were the only middle-income countries represented. Entire regions, including South Asia and Sub-Saharan Africa, were not represented in any of the existing evidence.

Across studies, PM was the most frequently studied air pollutant (six studies), followed by PM_2·5_ (three studies). All other air pollutants were examined in only one or two studies.

Study designs used to investigate the relationship between SRB and air quality were highly heterogeneous. Three studies used ecological, population-level approaches (Miraglia et al., 2013; Arima, 2023; Tsai, Weng and Yang, 2025). Two adopted quasi-experimental designs, with wildfire (Goin et al., 2024) or regulatory change used as an exogenous shock to air quality (Sanders and Stoecker, 2015).

### 3.3 Exposure and outcome measures

Table 2 summarises how air pollution exposures and the sex ratio at birth were measured. There was considerable variation in the temporal resolution of exposure assessment. Seven studies relied on aggregate-level data (Lin, Li and Mao, 2006; Miraglia et al., 2013; Sanders and Stoecker, 2015; Santoro et al., 2016; Houdek, Dvouletý and Pažitka, 2020; Arima, 2023; Tsai, Weng and Yang, 2025), typically assigning exposure based on annual births without aligning exposure windows to the gestational period. Two studies tracked exposure throughout pregnancy but summarised this into a cumulative average of exposure across the woman’s entire pregnancy (Candela et al., 2013; Ghosh et al., 2019), precluding analysis of critical windows of gestational exposure. Two studies assigned exposure with greater temporal specificity: one assessed daily exposure in the two weeks prior to conception (Lin et al., 2015), while another examined the timing of wildfire events relative to gestational age to identify critical windows of vulnerability (Goin et al., 2024).

Only four studies assigned exposure at the individual level (Candela et al., 2013; Santoro et al., 2016; Ghosh et al., 2019; Goin et al., 2024), typically using the mother’s residential postcode at birth and birth date to attribute air pollution levels during the pregnancy. The remaining studies attributed exposure at the area level, applying average pollution estimates across entire cities, districts, or counties (Lin, Li and Mao, 2006; Miraglia et al., 2013; Lin et al., 2015; Sanders and Stoecker, 2015; Houdek, Dvouletý and Pažitka, 2020; Arima, 2023; Tsai, Weng and Yang, 2025). Exposure was treated either as a continuous variable (for example, daily, monthly, or annual mean pollution levels) (Miraglia et al., 2013; Lin et al., 2015; Sanders and Stoecker, 2015; Ghosh et al., 2019; Houdek, Dvouletý and Pažitka, 2020; Arima, 2023; Tsai, Weng and Yang, 2025), categorical (e.g., quintiles) (Lin, Li and Mao, 2006; Candela et al., 2013; Santoro et al., 2016), or binary thresholds exceedances to distinguish exposed from unexposed areas (Goin et al., 2024).

### 3.4 Main findings

**Figure 3.**
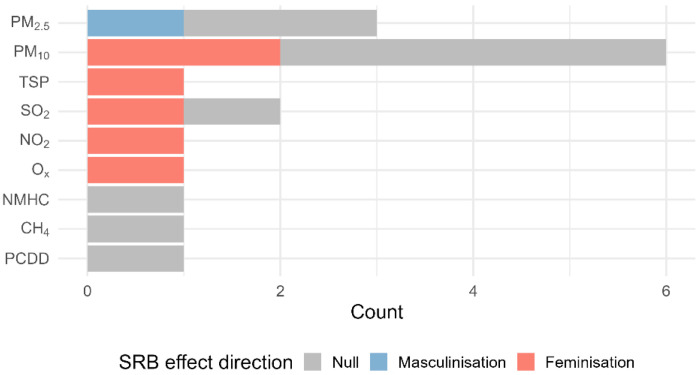
Effect direction counts

The main findings are presented in Table 3 (by study) and Figure 3 (by air pollutant type).

Four studies reported that higher levels of air pollution were associated with a feminisation of the SRB (Miraglia et al., 2013; Lin et al., 2015; Sanders and Stoecker, 2015; Arima, 2023). This association was observed for various pollutants, including PM_10_ (Miraglia et al., 2013; Lin et al., 2015), total suspended particulates (TSP) (Sanders and Stoecker, 2015), sulphur dioxide (SO) (Lin et al., 2015), nitrogen dioxide (NO) (Lin et al., 2015), and oxides (Arima, 2023). One study found that increased PM_2·5_ exposure was associated with a masculinisation of the SRB (Tsai, Weng and Yang, 2025).The remaining six studies reported only null findings, either for one or multiple pollutants (Lin, Li and Mao, 2006; Candela et al., 2013; Santoro et al., 2016; Ghosh et al., 2019; Houdek, Dvouletý and Pažitka, 2020; Goin et al., 2024, p. 202).

Eight studies adjusted for some individual-level confounders (Lin, Li and Mao, 2006; Candela et al., 2013; Lin et al., 2015; Sanders and Stoecker, 2015; Santoro et al., 2016; Ghosh et al., 2019; Houdek, Dvouletý and Pažitka, 2020; Goin et al., 2024), five of which also included area-level covariates. Only three studies included other climatic stressors (Lin et al., 2015; Sanders and Stoecker, 2015; Goin et al., 2024), such as temperature and humidity, which influence pollutant dispersion and intensity, as well as pregnancy outcomes. However, only one study accounted for maternal health status, including smoking patterns and maternal morbidity (Candela et al., 2013). Potential confounding variables such as maternal smoking and body mass index (BMI) were not adjusted for in the other ten studies, even though they may be associated with socioeconomic status, air pollution exposure, and potentially sex-biased pregnancy loss. Similarly, although two studies assessed industrial pollution alongside ambient exposures (Candela et al., 2013; Ghosh et al., 2019), none examined potential confounding from other sources such as indoor cooking fuels or occupational exposures.

### 3.5 Risk of bias

Risk of bias scores are presented in Table 3. On our modified 20-point scale, five studies were classified as high risk of bias (<10) (Miraglia et al., 2013; Santoro et al., 2016; Houdek, Dvouletý and Pažitka, 2020; Arima, 2023; Tsai, Weng and Yang, 2025), two as medium risk (11–15) (Lin, Li and Mao, 2006; Sanders and Stoecker, 2015), and four as low risk (16–20) (Candela et al., 2013; Lin et al., 2015, p. 20; Ghosh et al., 2019; Goin et al., 2024) (see appendix p.5 for score breakdown). The most common limitations were related to exposure misclassification, particularly in ecological studies that aggregated births within a given period (most commonly by calendar year) and measured air quality over the same period, without attributing exposure to the gestational window at the individual level. Additional concerns included weak causal inference due to study design limitations, and restricted sample representativeness, for instance when geographically restricted to women residing within a small radius of an MWI (Candela et al., 2013; Santoro et al., 2016; Ghosh et al., 2019).

### 3.6 Biological mechanisms

Across the 11 studies, the mechanisms through which exposure to ambient air pollution may affect the SRB were largely unexplored. Only one study, Lin et al (2015) explicitly examined maternal exposure in a defined pre-conception window, up to 13 days before the estimated conception date (Lin et al., 2015). Three studies assessed maternal exposure during pregnancy at the individual-level and inferred sex-differentials in early conceptus and pregnancy loss based on shifts in the SRB. However, two of these aggregated maternal exposure into a single summary measure, precluding the identification of windows of gestational vulnerability or the effect of chronic versus acute exposure (Candela et al., 2013; Ghosh et al., 2019). In the remaining seven studies, birth outcomes were aggregated over calendar periods without temporally aligning exposure to specific gestational windows, also limiting the ability to identify mechanisms underlying changes in the SRB (Lin, Li and Mao, 2006; Miraglia et al., 2013; Sanders and Stoecker, 2015; Santoro et al., 2016; Houdek, Dvouletý and Pažitka, 2020; Arima, 2023; Tsai, Weng and Yang, 2025).

## 4. Discussion

The SRB is a sentinel marker of reproductive stress at the population level, reflecting the cumulative impact of sex-biased conception and pregnancy losses that would otherwise remain largely unobserved. Despite growing public health concern about ambient air pollution, and the large proportion of the world’s population already exposed to air pollution that exceeds WHO standards (World Health Organization, 2021), its association with the SRB remains underexplored and poorly understood. To our knowledge, this is the first systematic review focused specifically on the contribution of air pollution to the SRB.

From the 11 studies included in our review, the evidence for the effect of air pollution on the SRB is limited, highly heterogeneous, and largely inconclusive. This finding is consistent with an existing review of environmental pollutants (Azizi et al., 2024). Most studies reported null findings, including those with the lowest risk of bias (Ghosh et al., 2019; Goin et al., 2024). Four studies found weak evidence of a feminisation of the SRB associated with exposure to ambient air pollution (Miraglia et al., 2013; Lin et al., 2015; Sanders and Stoecker, 2015; Arima, 2023), though these studies varied in their risk of bias. Feminisation of live births, resulting in a reduced SRB, is consistent with the hypothesis that male conceptuses are more likely to be spontaneously terminated under maternal stress (Bruckner and Catalano, 2018; Catalano, Casey and Bruckner, 2020). Evidence that ambient air pollution differentially increases male conceptus loss also supports the Trivers-Willard hypothesis, which predicts that adverse conditions favour the survival of female offspring (Trivers and Willard, 1973). However, heterogeneity in pollutant types, exposure windows, effect measures, and study design precluded meta-analysis to estimate the direction and strength of the association between air pollution and the SRB. There is an urgent need for new primary studies using standardised methods to improve comparability and facilitate future meta-analyses.

The existing evidence is geographically narrow, limited to only eight countries (Italy, China, Taiwan, Czech Republic, Brazil, the UK, Japan, and the USA) and lacks data from regions with some of the world’s poorest air quality and greatest burden of ambient air pollution attributable morbidity and mortality, including South Asia and sub-Saharan Africa.(WHO, 2025) Existing research also focuses mainly on particulate matter (PM_10_ and PM_2·5_), with minimal examination of other pollutants such as ozone (O), nitrogen oxides (NO), or sulphur dioxide (SO). This is a critical gap, as co-exposure to multiple pollutants may influence toxicity and reproductive health in complex ways (Chen et al., 2024; He et al., 2025). For instance, PM_2·5_ and O co-exposure may synergistic effects exceeding either pollutant alone (He et al., 2025), yet such interactions remain largely unexplored for pregnancy loss, including as indicated at the population-level by shifts in the SRB. Moreover, it is unclear which levels of air pollution are harmful for the SRB or whether associations follow a linear dose–response--information that is essential for policy design.

Methodological quality across the 11 studies was highly variable, five studies rated as having a high risk of bias. Many were limited by exposure timing, exposure misclassification, and omission of confounders. Seven studies used aggregate birth outcome data (Lin, Li and Mao, 2006; Miraglia et al., 2013; Sanders and Stoecker, 2015; Santoro et al., 2016; Houdek, Dvouletý and Pažitka, 2020; Arima, 2023; Tsai, Weng and Yang, 2025) (e.g., by year), without linking exposure to individual pregnancies, precluding temporal alignment and identification of critical windows of vulnerability. Among the four studies with exposure aligned with pregnancy timing, two still used cumulative averages across the entire gestation (Candela et al., 2013; Ghosh et al., 2019). Only two studies examined exposure with greater temporal specificity: one assessed daily exposure in the two weeks prior to conception (Lin et al., 2015), and another examined wildfire events relative to gestational age (Goin et al., 2024).

Potential exposure misclassification was also common. Seven studies relied on area-level exposure from fixed-site monitors, with accuracy depending on area size and spatial variability of pollutant concentrations (Lin, Li and Mao, 2006; Miraglia et al., 2013; Lin et al., 2015; Sanders and Stoecker, 2015; Houdek, Dvouletý and Pažitka, 2020; Arima, 2023; Tsai, Weng and Yang, 2025). Studies that estimated individual-level attribution with greater spatial specificity used air quality models that incorporated maternal residence and pollutant dispersion influenced by emission sources, distance, climate and topography (Candela et al., 2013; Santoro et al., 2016; Ghosh et al., 2019; Goin et al., 2024). However, these assumed a fixed residence throughout pregnancy and did not capture exposure occurring away from home. Misclassification may persist, particularly in high pollution settings where pregnant individuals modify mobility patterns to avoid harmful air.

Third, the omission of potential confounders was common. Key maternal health characteristics, including smoking and maternal morbidities, were omitted from all but one study (Candela et al., 2013). No study included body mass index (BMI). Maternal smoking (Parazzini et al., 2005; Beratis, Asimacopoulou and Varvarigou, 2008; Koshy et al., 2010) and low or high maternal BMI (Cagnacci et al., 2004; DeVilbiss et al., 2022) may correlate with deprivation and residence in more polluted areas as well as pregnancy loss. Only two studies controlled for seasonality (Lin et al., 2015; Ghosh et al., 2019), despite strong seasonal variation in both air pollution and births. Few studies addressed spatial confounding – only one used random effects (Ghosh et al., 2019, p. 201), another applied district fixed effects (Houdek, Dvouletý and Pažitka, 2020), and one employed a generalised synthetic control approach to address both time-varying and invariant confounding (Goin et al., 2024).

Finally, the role of heat in the association between air pollution and the SRB warrants further attention. With a few exceptions (Lin et al., 2015; Sanders and Stoecker, 2015; Goin et al., 2024), most studies did not consider temperature or explore how it should be treated in statistical models. Depending on causal direction, temperature could act as a confounder, a mediator, or an effect modifier. Interest in how heat and air pollution interact to affect health outcomes is growing, yet this issue remains largely unexamined in relation to the SRB.

### 4.1 Biological mechanisms

Across the 11 studies, biological mechanisms were underexplored and remain difficult to disentangle. Observed shifts in the SRB may reflect (1) sex-biased conception, influenced by maternal and/or paternal factors such as oocyte or sperm quality, or (2) sex-biased pregnancy loss, encompassing implantation failures as well as differential survival of male versus female conceptuses throughout gestation. The first pathway is challenging to investigate outside of assisted reproduction settings. Most population-based studies, which estimate maternal exposures retrospectively from live births, are better positioned to capture pregnancy loss mechanisms. Only one study explicitly examined pre-conception mechanisms (Lin et al., 2015), which may reflect a effects on sperm quality through oxidative DNA damage or epigenetic changes (James, 2001; Van Larebeke et al., 2008; Radwan et al., 2018). Three studies investigated post-conception exposures consistent with pregnancy loss mechanisms but did not identify specific biological pathways (Candela et al., 2013, p. 20; Ghosh et al., 2019; Goin et al., 2024). Air pollutants can trigger oxidative stress, resulting in DNA damage to gametes or embryonic tissues (Carré et al., 2017), they may interfere with endocrine signalling that is critical for sex-specific embryonic development; and they can impair placental function, which differs by foetal sex and may render male foetuses more susceptible to adverse intrauterine conditions (Clifton, 2010; Braun et al., 2022). For the remaining seven studies, they did not attempt to disentangle the contribution of conception effects and differential pregnancy loss on the SRB (Lin, Li and Mao, 2006; Miraglia et al., 2013; Sanders and Stoecker, 2015; Santoro et al., 2016; Houdek, Dvouletý and Pažitka, 2020; Arima, 2023; Tsai, Weng and Yang, 2025). Advancing understanding of these pathways is essential to interpret observed SRB changes and identify when, how, and in whom ambient air pollution affects reproductive outcomes.

### 4.2 Strengths and limitations

This systematic review has several strengths, including a comprehensive search strategy, double screening and extraction, and a conceptual framework linking ambient air pollution to the SRB. However, we could not conduct a meta-analysis or assess publication bias due to the limited, heterogeneous evidence base, underscoring the need for additional primary research. Future studies should prioritise: (1) identifying critical windows of exposure; (2) using individual-level data with accurate exposure measures and key confounders (e.g. seasonality and place specific characteristics); (3) expanding studies to high-pollution, underrepresented regions, especially sub-Saharan Africa and South Asia; (4) incorporating pollutant combinations and investigating the role of heat as an effect modifier, modifier or confounder; and (5) directly investigating biological mechanisms through biomarkers of oxidative stress, placental function, and endocrine disruption.

As ambient air quality deteriorates in a rapidly warming planet, understanding how air pollution affects the SRB is increasingly important. As a sentinel indicator of reproductive health, the SRB captures the otherwise unobserved, cumulative impact of sex-biased conception and pregnancy loss. Strengthening the evidence on this association is critical to inform air quality regulation and guide public health responses that protect reproductive health.

## Supplementary Material

Supplement 1

## Figures and Tables

**Table 1:** Study characteristics

Study	Study location	Sample detail	Observation period	Sample size births	Type of study	Pollutant source	Pollutants assessed
Arima et al (2023) ^1^	Fukue Island, Japan	Not reported	Pollutants: 2013-2022 Births: 2014- 2022	1,835	Ecological (population-level, time-series)	Ambient	PM_2·5_, SO_2_, OX
Candela et al (2013) ^2^	Emilia-Romagna, Italy	4km radius around eight incinerators	2003-2010	21,164	Observational (individual-level, retrospective cohort)	Municipal waste incinerators (MWI)	PM_10_
Ghosh et al (2019) ^3^	Great Britain	22 municipal waste incinerators	2003-2010	1,025,064	Matched case control (individual-level)	MWI	PM_10_
Goin et al (2024) ^4^	Paradise, California, USA	Not reported	2017-2019	Not reported	Quasi-experimental (individual-level, natural experiment)	Wildfire	PM_2·5_
Houdek et al (2020) ^5^	Czech Republic	Residents of 216 districts	1992-2010	446,411	Observational (individual-level, retrospective cohort)	Ambient	PM_10_
Lin et al (2006) ^6^	Taipei, Taiwan	One MWI	1991, 1997	1991: 6,697 1997: 6,282	Observational (ecological exposure, individual-level covariates, cross-sectional pre-post comparison)	MWI	PCDDs
Lin et al (2015) ^7^	Guangzhou, China	Births in one major maternity hospital	2006-2011	81,711	Observational: (individual-level, retrospective cohort)	Ambient	PM_10_, NO_2_, SO_2_
Miraglia et al (2013) ^8^	Sao Paulo, Brazil	-	2000-2007	53,612	Ecological (population-level, time-series)	Ambient	PM_10_
Sanders & Stoecker (2015) ^9^	USA	198 counties, 35 states	1970-1973	2,709,415	Quasi-experimental (population-level, natural experiment)	Ambient	TSPs
Santoro et al (2016) ^10^	Arezzo, Tuscany, Italy	One incinerator plant	2001-2010	3,069	Observational (individual-level, retrospective cohort)	MWI	PM_10_
Tsai et al (2025) ^11^	Taipei, Taiwan	Not reported	1992-2023	Period 1: 137,438 Period 2: 263,016 Period 3: 261,708 Period 4: 163,630	Ecological (population-level, time series)	Ambient, pre-post implementation of a rapid transit system	PM_2·5_

**Table 2:** Exposure and outcome measures

Study	Exposure window (in relation to pregnancy)	Frequency of exposure measurement throughout pregnancy	Spatial inclusion criteria	Exposure attribution	AQ definition	Available temporal frequency of AQ data	Sex ratio outcome measure
Arima et al (2023)^1^	Year of birth	NA (gestational window not measured)	Not specified	Area-level	Monthly mean exposure during daylight hours	Hourly aggregated to monthly	Male-to-female ratio at birth
Candela et al (2013) ^2^	Entire pregnancy	Summary average over pregnancy	Mothers’ residence within 4km radius around MWI	Individual-level	Categorical pregnancy-average, quintiles	Half-hourly (~17,000 measures/year)	Proportion female births (female / total)
Ghosh et al (2019) ^3^	Entire pregnancy	Summary average over pregnancy	Mothers’ residence within 10km radius around MWI	Individual-level	Mean daily exposure across pregnancy	Daily aggregated to pregnancy average	Female-to-male ratio at birth
Goin et al (2024) ^4^	Dates of wildfire during pregnancy	Weekly	Mothers’ residence within census block group centroid and plume polygons	Individual-level	Binary exposure (thresholds exceeded and duration)	Hourly aggregated to daily	Male-to-female ratio at birth
Houdek et al (2020) ^5^	Month of birth	Summary average over month of birth	District-level (216 stations)	Area-level	Monthly average	Daily aggregated to monthly	Binary outcome: male birth
Lin et al (2006) ^6^	Year of birth	NA (gestational window not measured)	Not specified	Area-level	Categorical annual exposure (reference, medium, high)	Hourly aggregated to annual	Binary outcome: female birth
Lin et al (2015) ^7^	13 days pre-conception	Daily	City-wide average (9 monitors)	Area-level	Daily average	Daily	Binary outcome: female birth
Miraglia et al (2013) ^8^	Year of birth	NA (gestational window not measured)	City-wide average (9 monitors)	Area-level	Annual average	Daily aggregated to annual	Proportion male births (male / total)
Sanders & Stoecker (2015) ^9^	Year of birth	NA (gestational window not measured)	County-level	Area-level	Annual exposure (attainment status)	Daily aggregated to annual	Proportion male births (male / total)
Santoro et al (2016) ^10^	Year of birth	NA (gestational window not measured)	Mothers’ residence within 12km around MWI	Individual-level	Categorical annual exposure (low/medium/high)	Annual	Male-to-female birth ratio
Tsai et al (2025) ^11^	Year of birth	NA (gestational window not measured)	City-wide average (6 monitors)	Area-level	Annual average PM2.5 (estimated from PM10)	Hourly data aggregated to daily and annual	Proportion male births (male / total)

**Table 3:** Main results and risk of bias

Study	Pollutant type and direction of effect^1^									Effect measure	Effect size	Adjustment for confounders	Risk of bias^2^
	PM_2.5_	PM_10_	TSP	SO_2_	NO_2_	OX	NMHC	CH_4_	PCDD	Linear regression beta coefficient	β coefficient: −0.311 (95% CI: −0.516, −0.106)		
Arima et al (2023)	∅			∅		♀	∅	∅		Adjusted odds ratio	ORs (quintiles 2–5 vs 1): 0.93, 0.95, 1.00, 0.91Test for trend: p = 0.249	None	High (9/20)
Candela et al (2013)		∅								Adjusted odds ratio	OR for doubling: 1.00 OR per km from MWI: 1.00	Individual-level: maternal age, birth order, maternal nationality, maternal education, exposure to other pollution sources (NOx), time period, maternal smoking, previous hospitalisations	Low (16/20)
Ghosh et al (2019)		∅								Average treatment effect (ATT)	No significant associations Highest exposure (PM2.5 > 50 for 7 days): ATT = 0.014 (−0.025 to 0.053)	Individual-level: year of birth, sex, season of birth, maternal age, deprivation, ethnicity Area-level: socio-economic status, population density, percentage of non-white; other potential sources of PM10 - local road density and number of industries within 10km; Random effects	Low (18/20)
Goin et al (2024)	∅									Adjusted odds ratio	β coefficient = 0.0000123 (SE 0.000194) OR = 1.0000123	Individual-level: maternal education, unemployment Area: temperature Synthetic control with fixed effects helped account for time-varying confounding	Low (19/20)
Houdek et al (2020)		∅								Adjusted odds ratio	ORs and CIs: 1991: >0.05 pg TEQ/m3: 1.00 (95% CI: 0.86–1.16) 0.03–0.05pg TEQ/m3: 0.95 (0.85–1.05) 1997: >0.05 pg TEQ/m3: 0.90 (95% CI: 0.78–1.05) 0.03–0.05pg TEQ/m3: (95% CI: 0.95 0.86–1.07)	Individual-level: citizenship, marital status, educational level, maternal age, father’s education Area-level: property prices Year and district fixed effects	High (6/20)
Lin et al (2006)									∅	Excess risk from adjusted odds ratio	ER for PM10 (lag 2) AOR: 0.64% (0.35%, 0.93%) ER for SO2 (lag 3) AOR: 0.35% (95% CI: 0.11%, 0.60%) ER for NO2 (lag 3) AOR: 0.91% (95% CI: 0.27%, 1.56%)	Individual-level: maternal age, educational level, birth order	Medium (11/20)
Lin et al (2015)		♀		♀	♀					Relative risk	β coefficient = −0.001 RR = 0.999	Individual-level: maternal age, maternal and paternal education, season of conception Area-level: previous 7 days’ mean temperature, relative humidity Smoothing splines for long-term trends	Low (16/20)
Miraglia et al (2013)		♀								Linear regression beta coefficient	Diff-in-diff estimate: −0.004 (0.004) IV estimate: −0.041 (p < 0.05) Clean Air Act impact: −0.47 percentage points	None	High (7/20)
Sanders & Stoecker (2015)			♀							Adjusted odds ratio	Medium: 1.07 (95% CI: 0.89–1.29) High: 1.17 (95% CI: 0.89–1.52) Trend: 1.08	Individual-level: child’s race, institutional delivery, skilled attendance at birth, birth order, maternal age, maternal education. Area-level: per capita income, total government transfer payments, employment level; annual average daily maximum temperature, annual precipitation	Medium (14/20)
Santoro et al (2016)		∅								Odds ratio	Period 1: 1.00 Period 2: 0.98 (95% CI: 0.97–0.99) Period 3: 0.97 (95% CI: 0.96–0.98) Period 4: 0.96 95% CI: (0.95–0.98 P for linear trend < 0.01”	Individual-level: maternal age, maternal education, birth order, country of origin, socioeconomic status	High (9/20)
Tsai et al (2025)	♂									Linear regression beta coefficient	β coefficient: −0.311 (95% CI: −0.516, −0.106)	None	High (7/20)

1 PM2.5: Particulate Matter <2.5 μm; PM10: Particulate Matter <10 μm; TSP: Total Suspended Particles; SO_2_: Sulphur Dioxide; NO_2_: Nitrogen Dioxide; OX: Oxidants (ozone and nitrogen dioxide); NMHC: Non-Methane Hydrocarbons; CH_4_: Methane; PCDD: Polychlorinated Dibenzo-p-Dioxins.

2 Risk of bias score out of (n/20); the higher the score, the lower the risk of bias. High risk: 0-10; medium risk: 11-15; low risk: 16-20. Study quality was appraised using an adapted version of the NIH Quality Assessment Tool for Observational Cohort and Cross-Sectional Studies (NHLBI 2014), modified to accommodate ecological, quasi-experimental, and time-series designs (see Appendix pp.4-5).

∅: Null effect on SRB with increased pollutant exposure

♀: Feminisation of the SRB with increased pollutant exposure

♂: Masculinisation of the SRB with increased pollutant exposure

## Data Availability

N/A all studies are available in the public domain.
